# Arteriogenesis and Therapeutic Angiogenesis in Its Multiple Aspects

**DOI:** 10.3390/cells9061439

**Published:** 2020-06-10

**Authors:** Elisabeth Deindl, Paul H. A. Quax

**Affiliations:** 1 Walter-Brendel-Centre of Experimental Medicine, University Hospital, Ludwig-Maximilians-University, 81377 Munich, Germany; 2 Department of Surgery, Einthoven Laboratory for Experimental Vascular Medicine, Leiden University Medical Center, 2300 RC Leiden, The Netherlands

Arteriogenesis, also frequently called collateral formation or even therapeutic angiogenesis, comprises those processes that lead to the formation and growth of collateral blood vessels that can act as natural bypasses to restore blood flow to distal tissues in occluded arteries. Both in coronary occlusive artery diseases as well as in peripheral occlusive arterial disease, arteriogenesis may play an important role in the restoration of blood flow. Despite the big clinical potential and the many promising clinical trials on arteriogenesis and therapeutic angiogenesis, the exact molecular mechanisms involved in the multifactorial processes of arteriogenesis are still not completely understood. In this inflammatory-driven vascular remodeling process, many cell types, both vascular cells and immune cells, many cytokines and growth factors, as well as various noncoding RNAs may be involved. Consequently, many questions regarding the exact molecular mechanisms involved in the regulation of the arteriogenic response still need to be answered, and these answers will contribute to defining new therapeutic options.

This Special Issue of *Cells* is devoted to all aspects of arteriogenesis, collateral formation and therapeutic angiogenesis. It contains articles that collectively provide a balanced, state-of-the-art view on various aspects of arteriogenesis and the underlying regulation of vascular remodeling.

As indicated above, arteriogenesis is an inflammatory-driven vascular remodeling process and Toll-like receptors (TLRs), especially TLR4, are known to be involved in arteriogenesis. Troidl et al., demonstrate that after the induction of hind limb ischemia in mice, the lipopeptide and TLR2/6 ligand macrophage-activating protein of 2-kDA (MALP-2) increased the growth of pre-existing collateral arteries in the upper hind limb, along with intimal endothelial cell proliferation in the collateral wall and pericollateral macrophage accumulation. In addition, MALP-2 increased capillary density in the lower hind limb. These promising results with the TLR2/6 ligand MALP-2 illustrate the potential to promote peripheral blood flow recovery by collateral artery growth by enhancing the inflammatory response [1].

The role of inflammation and immune cells in arteriogenesis is also illustrated by Bot el al., who extended the studies on the role of mast cells in arteriogenesis and collateral formation and demonstrated that local mast cell activation increased blood flow through the hind limb, due an increase in the diameter of the collaterals, as well as in the number of CD31^+^ capillaries. Together, these data illustrate that locally activated mast cell contribute to arteriogenesis and angiogenesis [2].

The induction of angiogenesis by vascular endothelial growth factor (VEGF) is well established, and the VEGF stimulation of endothelial cells encompasses a complex series of events that include the activation of various intracellular signaling cascades. Of these, the activation of ERK1/2 has been directly linked to the extent of arteriogenesis. Little is known about the individual contribution of ERK isoforms to this process. Ricard et al., focused on the role of ERK1/2 isoforms in adult arteriogenesis. The induction of acute hind limb ischemia resulted in excessive but poorly functional arteriogenesis in mice with a global deletion of Erk1, whereas mice with an endothelial-specific deletion of Erk2 exhibited a decreased arteriogenesis. They generated a floxed ERK1 mouse line and conditionally deleted the gene in macrophages, endothelial, and smooth muscle cells. While the endothelial or macrophage deletions of ERK1 failed to recapitulate the phenotype of the ERK1^−/−^ mice, the combined deletion of Erk1 in endothelial cells and macrophages came close to the phenotype in global Erk1 null mice. This shows that endothelial and macrophage ERK1 is critical to endothelial/macrophage crosstalk and effective adult arteriogenesis [3].

The importance of smooth muscle cell (SMC) proliferation in arteriogenesis is demonstrated by Lasch et al., They investigated the functional relevance of the potassium channels K_V_1.3 and K_Ca_3.1 for SMC proliferation in arteriogenesis and showed convincingly that the modulation of the potassium channel K_V_1.3 contributes to SMC proliferation in arteriogenesis, whereas K_Ca_3.1 is more likely to be involved in vasodilation [4].

VEGF is a key factor for endothelial cell proliferation and migration, as well as recruitment of pericytes and vessel assembly. VEGF can be modulated in many ways. In this Special Issue, Uslu et al., study the effects of FSAP (factor-VII-activating protease) on VEGF. The stimulatory effects of VEGF_165_ on endothelial cell proliferation, migration, and signal transduction were not altered by FSAP (factor-VII-activating protease) in vitro. However, FSAP inhibited VEGF_165_-mediated angiogenesis in the matrigel model in vivo, showing the role of the environment of growth-factor-mediated neovascularization [5].

Hypoxia and the (lack of) HIF1α degradation by the von Hippel-Lindau (VHL) protein complex are key determinants for VEGF activity in neovascularization. Lei et al., demonstrate very elegantly how the VHL/miR-212/miR-132 axis can play a crucial role the control of angiogenesis and that a scarcity of functional pVHL induces excessive vascular outgrowth, which is further enhanced by miR-212/132 expression, providing an exciting target for the modulation of angiogenesis [6].

MicroRNAs are small noncoding RNAs that post-transcriptionally regulate the expression of groups of target genes. However, these microRNAs can be modified themselves too, with all related consequences for processes they regulate like arteriogenesis and angiogenesis. Recent studies have revealed that many microRNAs have variants with altered terminal sequences, known as isomiRs. Additionally, endogenous microRNAs have been identified that carry biochemically modified nucleotides, revealing a dynamic microRNA epitranscriptome. Van der Kwast et al., provide in this Special Issue an overview on the mechanisms of how both types of microRNA alterations are dynamically regulated in response to ischemia and are able to influence angiogenesis and arteriogenesis [7].

The impact of hypoxia is studied by Parma et al., in their studies on intraplaque angiogenesis in lesion in murine vein grafts that are hypoxic and show profound angiogenesis in the plaque. Resolving the hypoxia by treatment of the mice with carbogen gas (95% oxygen) only had a short effect on the hypoxia in the tissue. However, this study demonstrates that long-term carbogen treatment did improve vein graft patency and plaque stability and reduced intraplaque macrophage accumulation via ROS-mediated DNA damage and apoptosis, but failed to have long-term effects on hypoxia and intraplaque angiogenesis [8].

The relation of atherosclerosis and the microvasculature is discussed in the review paper by Ziegler et al., in this Special Issue. They describe how atherosclerotic risk factors have their impact on capillary networks and that this is an element that is frequently forgotten in the current therapeutic revascularization strategies. They advocate that the microcirculatory changes during atherosclerosis, such as capillary rarefaction, warrant further investigation [9].

In the review by Caicedo et al., evidence for the involvement of the proangiogenic hormones of the growth hormone (GH)/IGF-I axis in arteriogenesis dealing with the arterial occlusion and making of them a potential therapy is described. All the elements that trigger the local and systemic production of GH/IGF-I, as well as their possible roles both in physiological and pathological conditions, are analyzed. Moreover, they describe the use of GH in the GHAS trial, in which GH or a placebo were administrated to patients suffering from critical limb ischemia with no option for revascularization [10].

Exercise training is the most promising and is the first step in the treatment of patients with peripheral arterial diseases. In their paper, Vogel et al., describe exercise-induced vascular adaptations under pathologically reduced blood flow and compare this to changes after artificially reduced blood flow. Major similarities include the overall ischemic situation, the changes in microRNA (miRNA) expression, and the increased production of nitric oxide synthase (NOS) with their associated arteriogenesis after training with blood flow restriction [11].

Last but not least, we address a specific form of arteriogenesis in this Special Issue. A huge collateral network protects the central nervous system from ischemia. Patients are at risk of spinal cord ischemia during (endovascular) aortic aneurysm repair surgery. However, predicting which patient will develop postoperative problems is difficult. One possible reason for this is the rather unknown arteriogenesis of the spinal cord blood supply. The review of Simon et al., aims to illuminate arteriogenesis in general, with the focus on the special needs of the spinal cord blood supply [12].

We believe that the papers in this Special Issue, each addressing a specific aspect of arteriogenesis and therapeutic angiogenesis, will help us to better understand the underlying mechanisms and will help to promote arteriogenesis and therapeutic angiogenesis effectively in patients with vascular occlusive diseases.
